# Supplementary figures and images for: A myristoyl switch at the plasma membrane triggers cleavage and oligomerization of Mason-Pfizer monkey virus matrix protein
Source: eLife. 2024 Mar 22;13:e93489. doi: 10.7554/eLife.93489 (PMC11014724; doi:10.7554/eLife.93489)

|                |   |    |   |   |   |    |   |   |   |    |
|----------------|---|----|---|---|---|----|---|---|---|----|
| M-PMV protease | - | -  | + | + | + | +  | + | + | + | +  |
| liposomes      | - | -  | - | - | - | -  | + | + | + | +  |
| time (h)       | 1 | 24 | 1 | 2 | 4 | 24 | 1 | 2 | 4 | 24 |

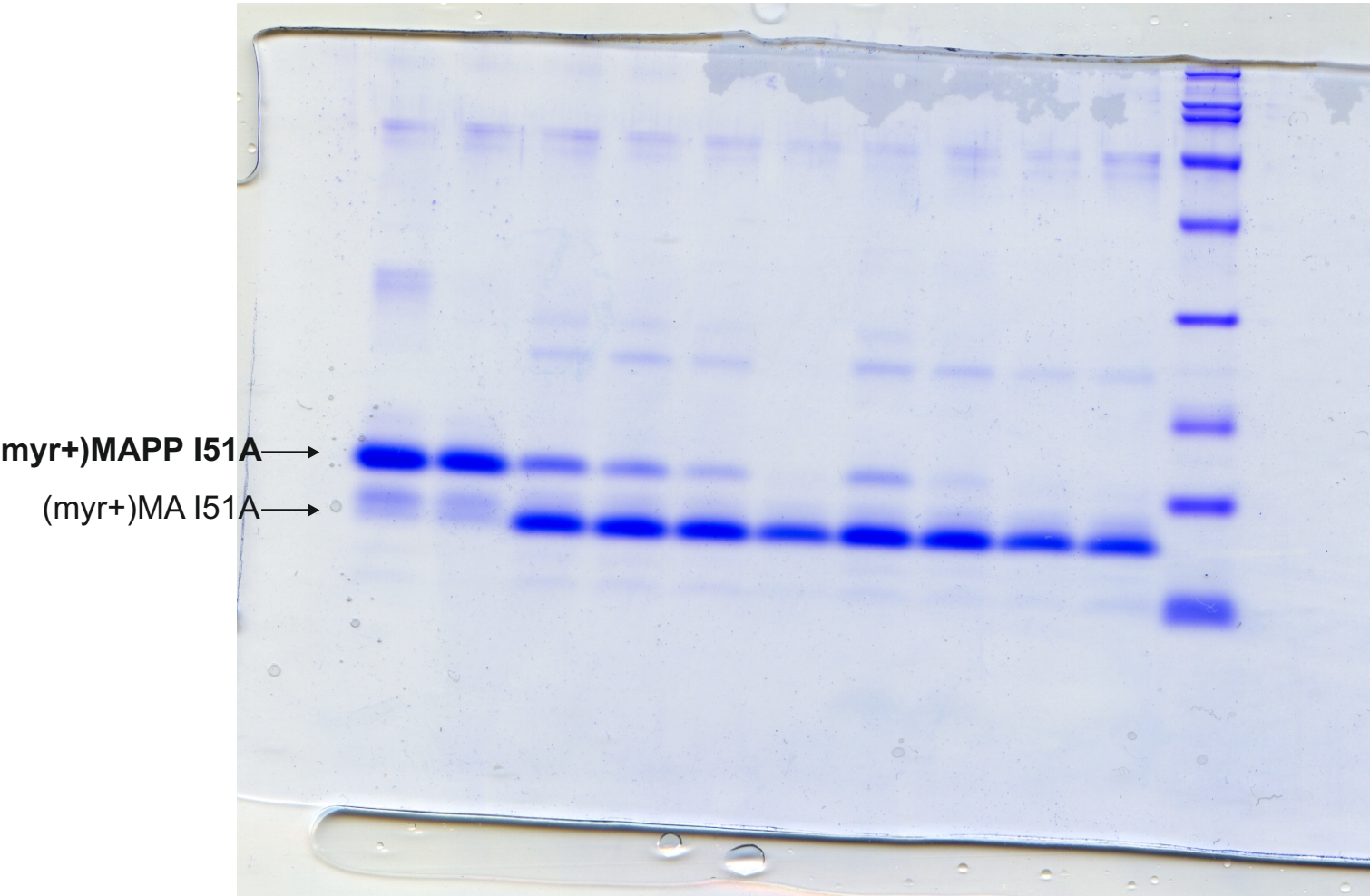

Supplement: Figure 1—source data 1. [file elife-93489-fig1-data1.zip › Figure 1- source data 1 Related to Figure 1C.pdf]

|                |   |    |   |   |   |    |   |   |   |    |
|----------------|---|----|---|---|---|----|---|---|---|----|
| M-PMV protease | - | -  | + | + | + | +  | + | + | + | +  |
| liposomes      | - | -  | - | - | - | -  | + | + | + | +  |
| time (h)       | 1 | 24 | 1 | 2 | 4 | 24 | 1 | 2 | 4 | 24 |

(myr+)MAPP A79V

→

(myr+)MA A79V

→

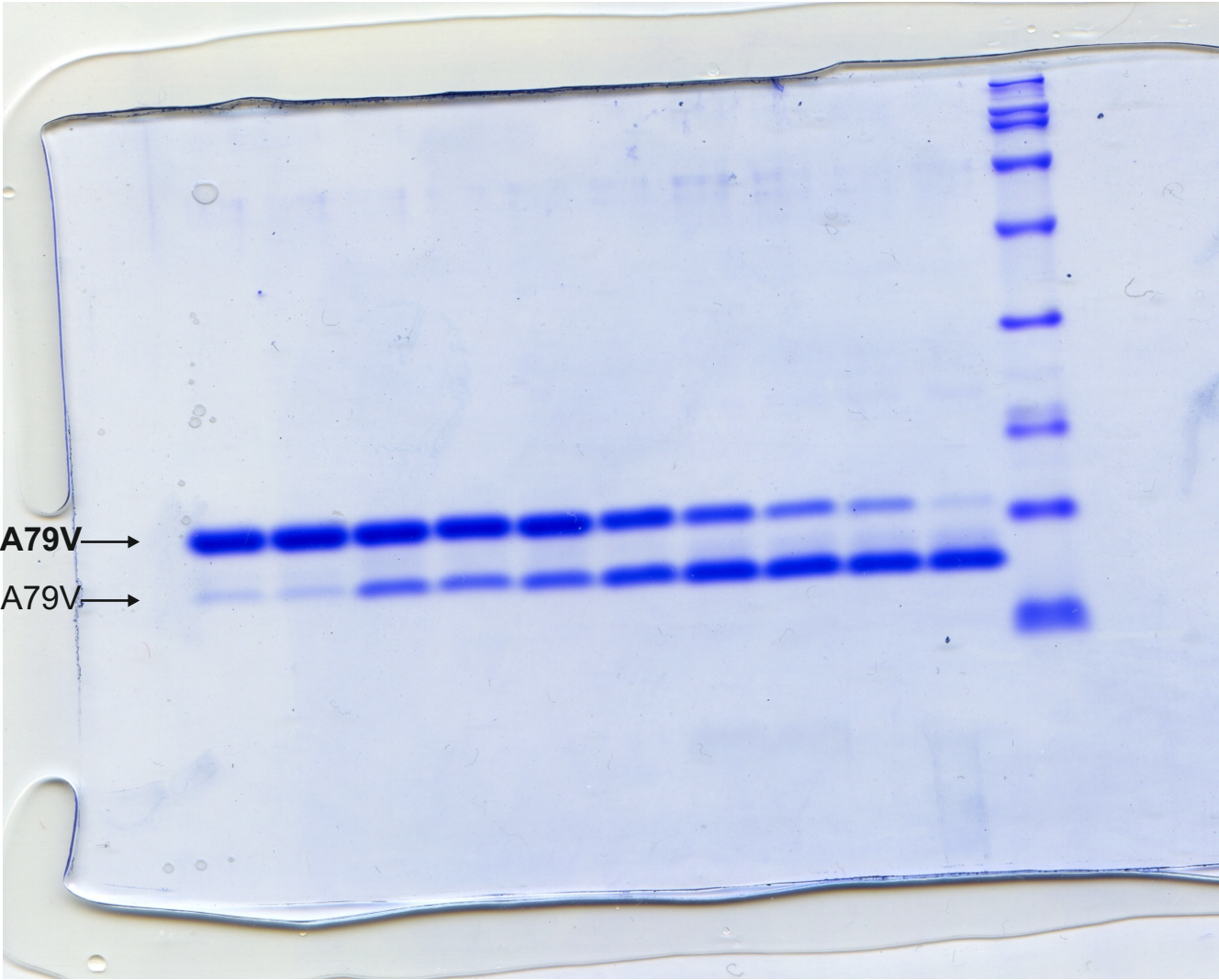

Supplement: Figure 1—source data 1. [file elife-93489-fig1-data1.zip › Figure 1- source data 1 Related to Figure 1D.pdf]

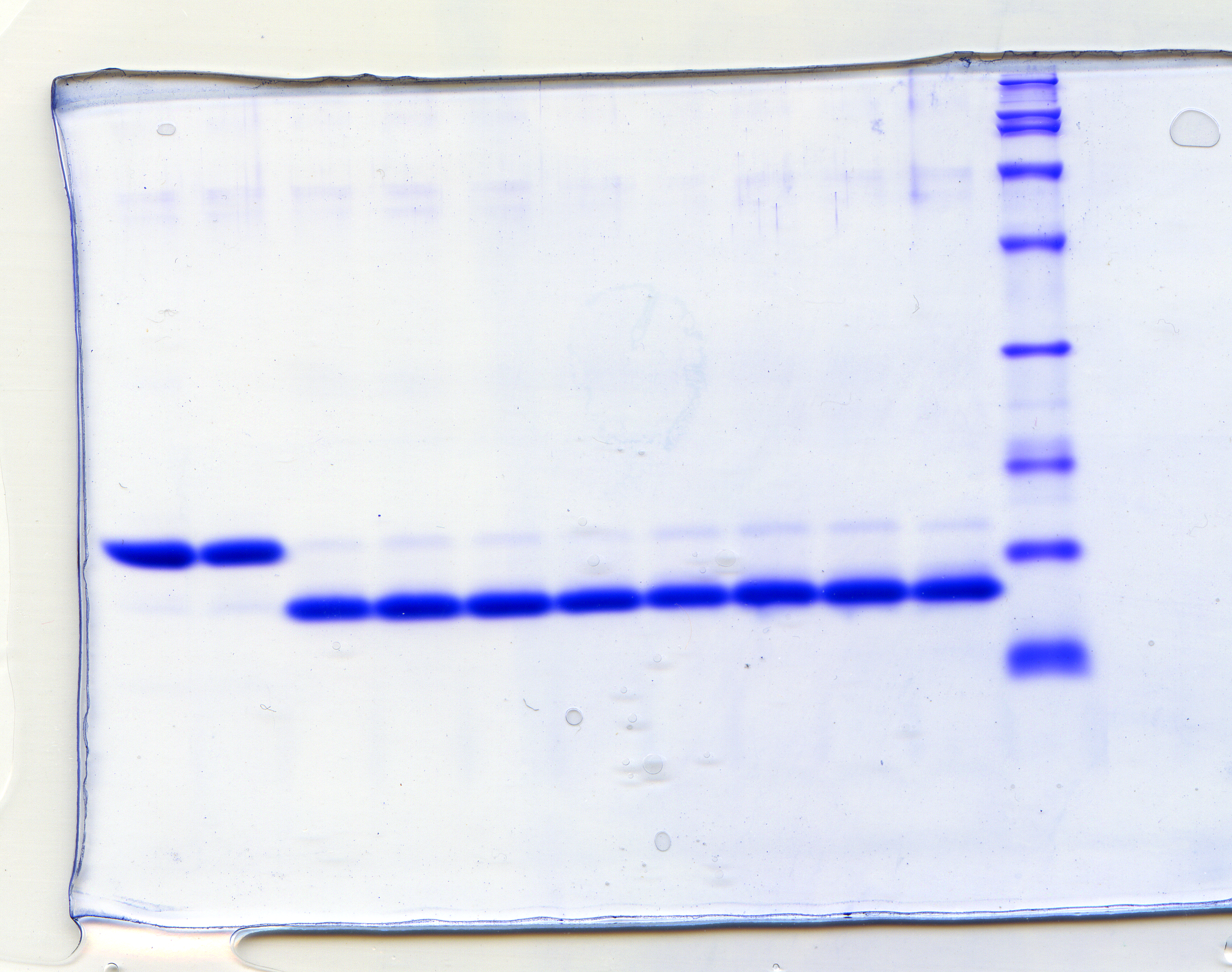

Supplement: Figure 1—source data 1. [file elife-93489-fig1-data1.zip › Figure 1- source data 2 Related to Figure 1A.tif]

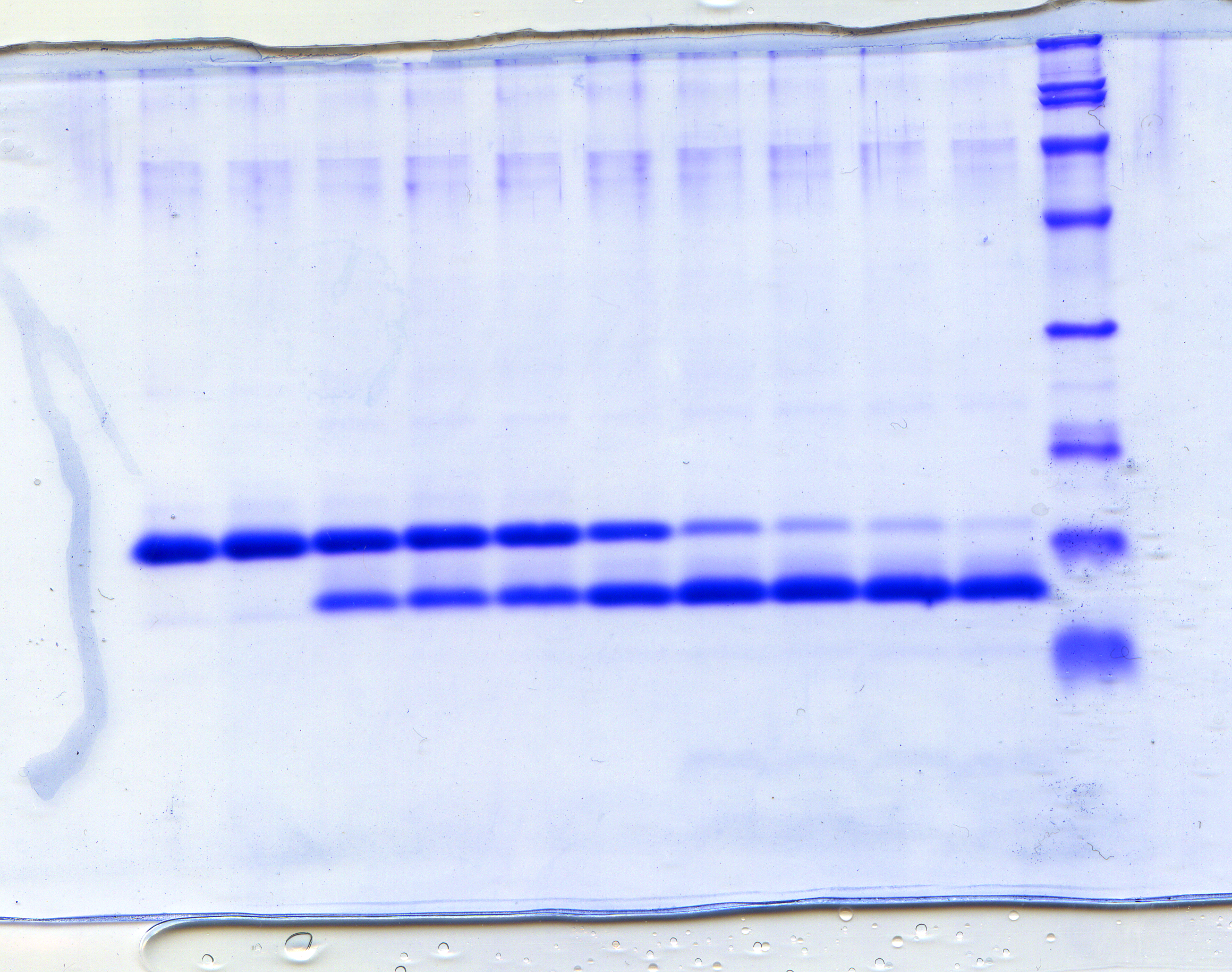

Supplement: Figure 1—source data 1. [file elife-93489-fig1-data1.zip › Figure 1- source data 2 Related to Figure 1B.tif]

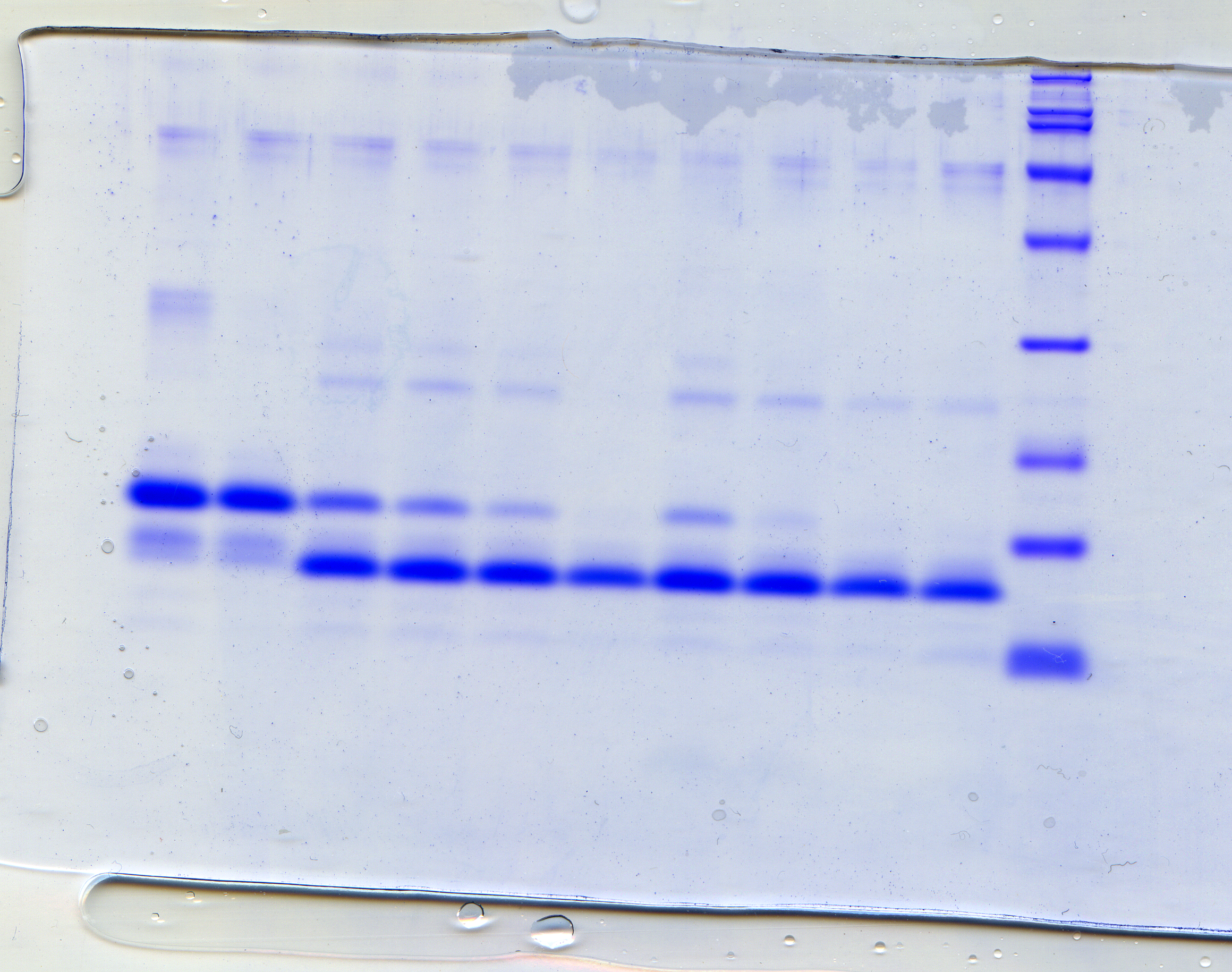

Supplement: Figure 1—source data 1. [file elife-93489-fig1-data1.zip › Figure 1- source data 2 Related to Figure 1C.tif]

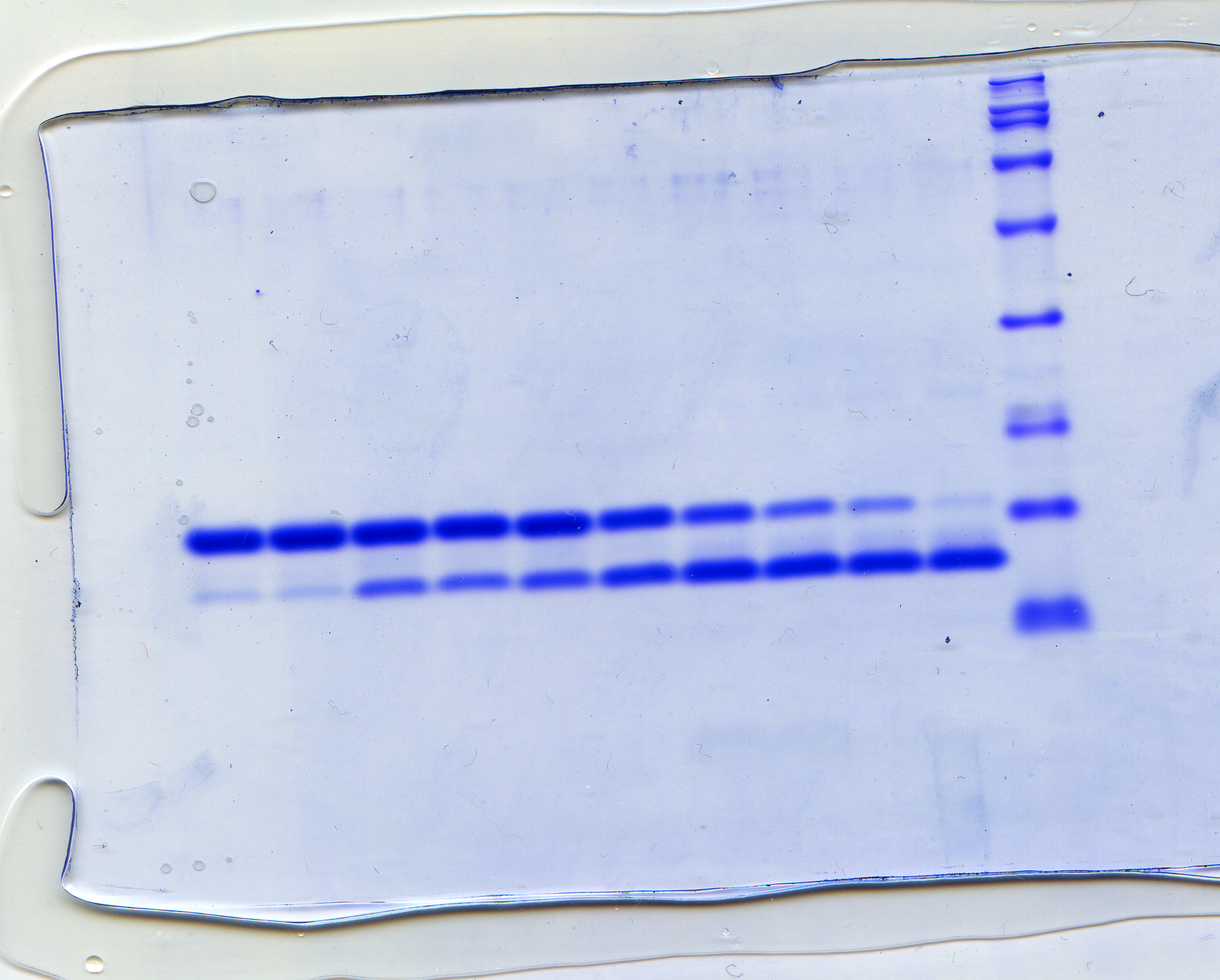

Supplement: Figure 1—source data 1. [file elife-93489-fig1-data1.zip › Figure 1- source data 2 Related to Figure 1D.tif]

|                |   |    |   |   |   |    |   |   |   |    |
|----------------|---|----|---|---|---|----|---|---|---|----|
| M-PMV protease | - | -  | + | + | + | +  | + | + | + | +  |
| liposomes      | - | -  | - | - | - | -  | + | + | + | +  |
| time (h)       | 1 | 24 | 1 | 2 | 4 | 24 | 1 | 2 | 4 | 24 |

(myr-)MAPP→

(myr-)MA→

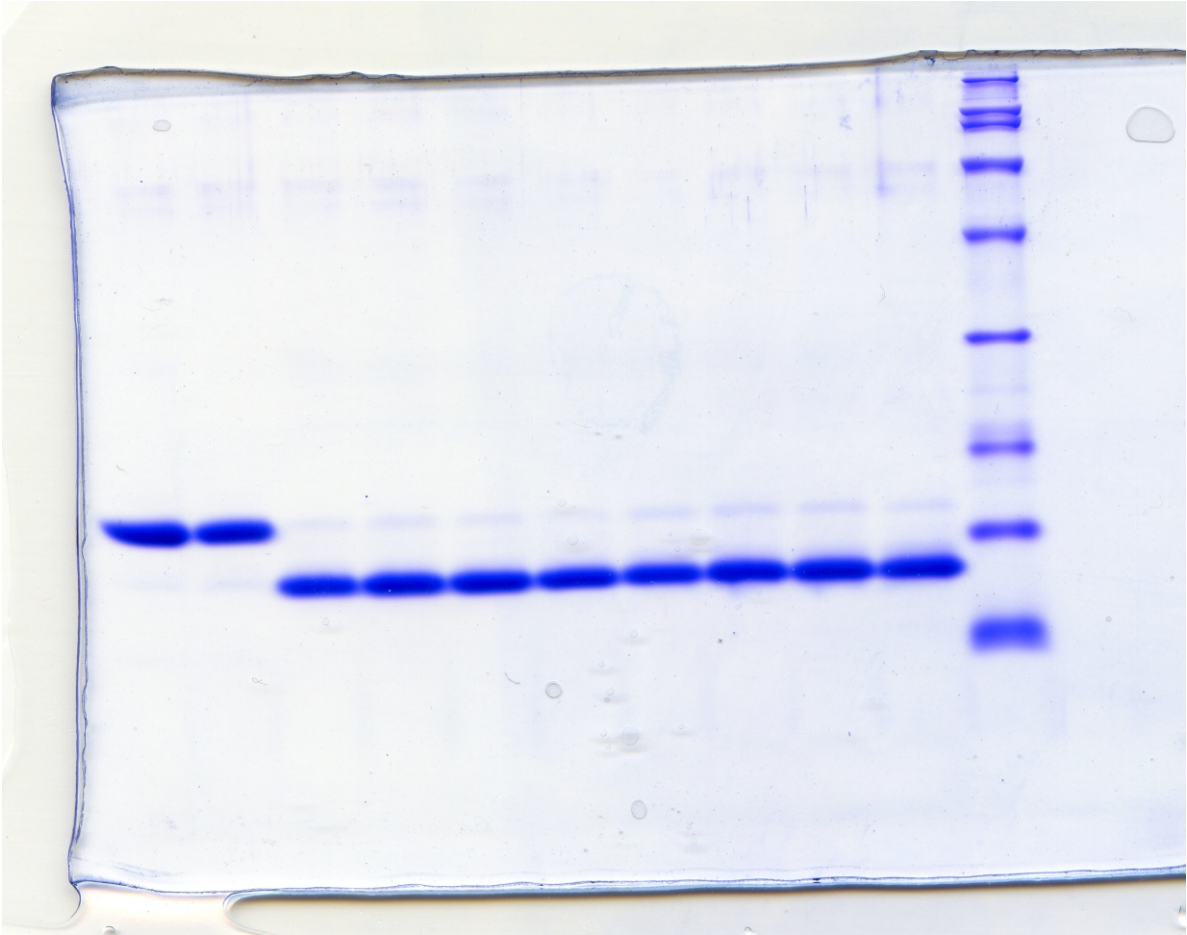

Supplement: Figure 1—source data 1. [file elife-93489-fig1-data1.zip › Figure 1- source data 1 Related to Figure 1A.pdf]

|                |   |    |   |   |   |    |   |   |   |    |
|----------------|---|----|---|---|---|----|---|---|---|----|
| M-PMV protease | - | -  | + | + | + | +  | + | + | + | +  |
| liposomes      | - | -  | - | - | - | -  | + | + | + | +  |
| time (h)       | 1 | 24 | 1 | 2 | 4 | 24 | 1 | 2 | 4 | 24 |

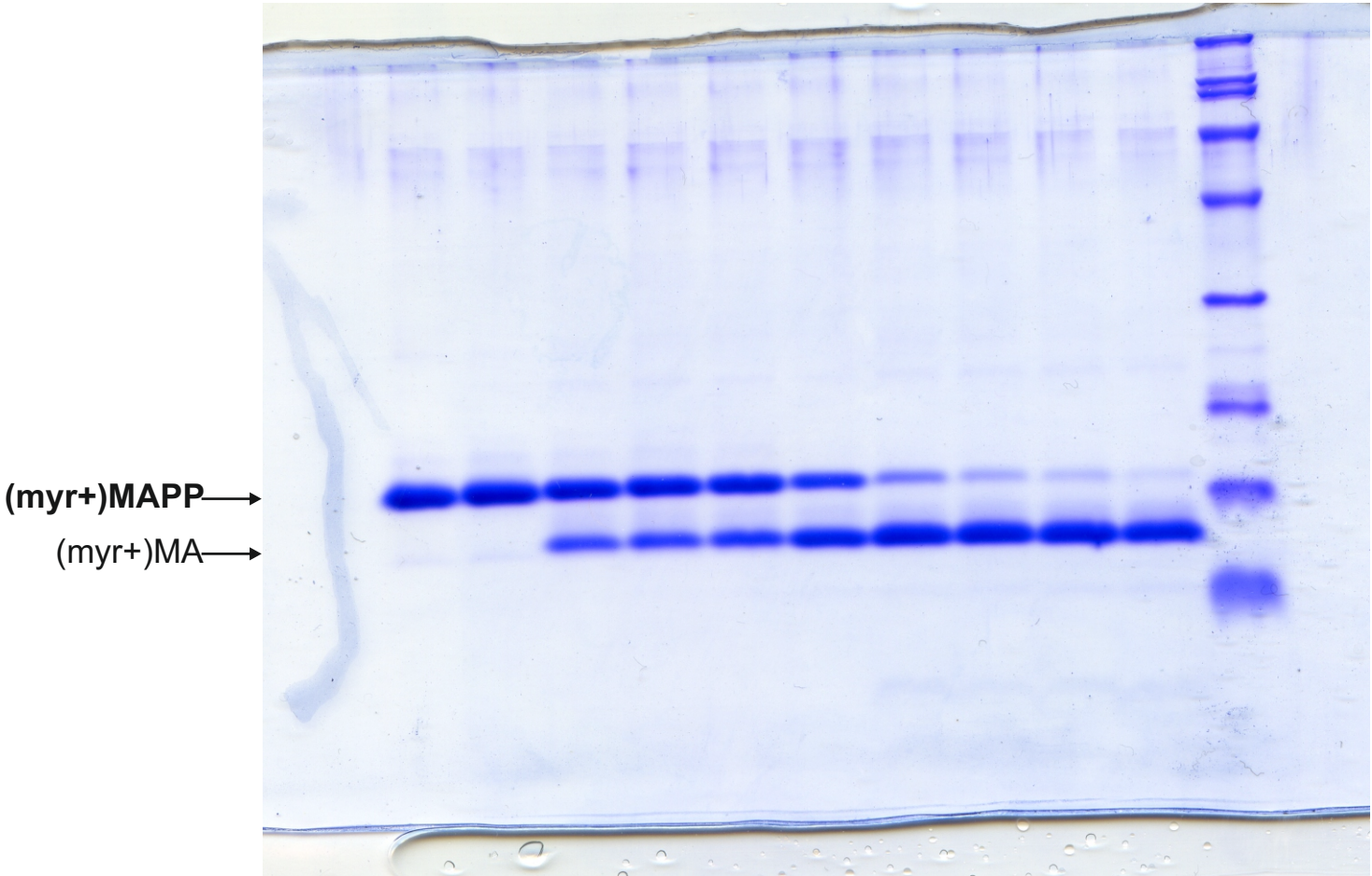

Supplement: Figure 1—source data 1. [file elife-93489-fig1-data1.zip › Figure 1- source data 1 Related to Figure 1B.pdf]

|           |           |   |           |   |
|-----------|-----------|---|-----------|---|
| dialysis: | non-red   |   | red       |   |
|           | liposomes |   | liposomes |   |
|           | -         | + | -         | + |

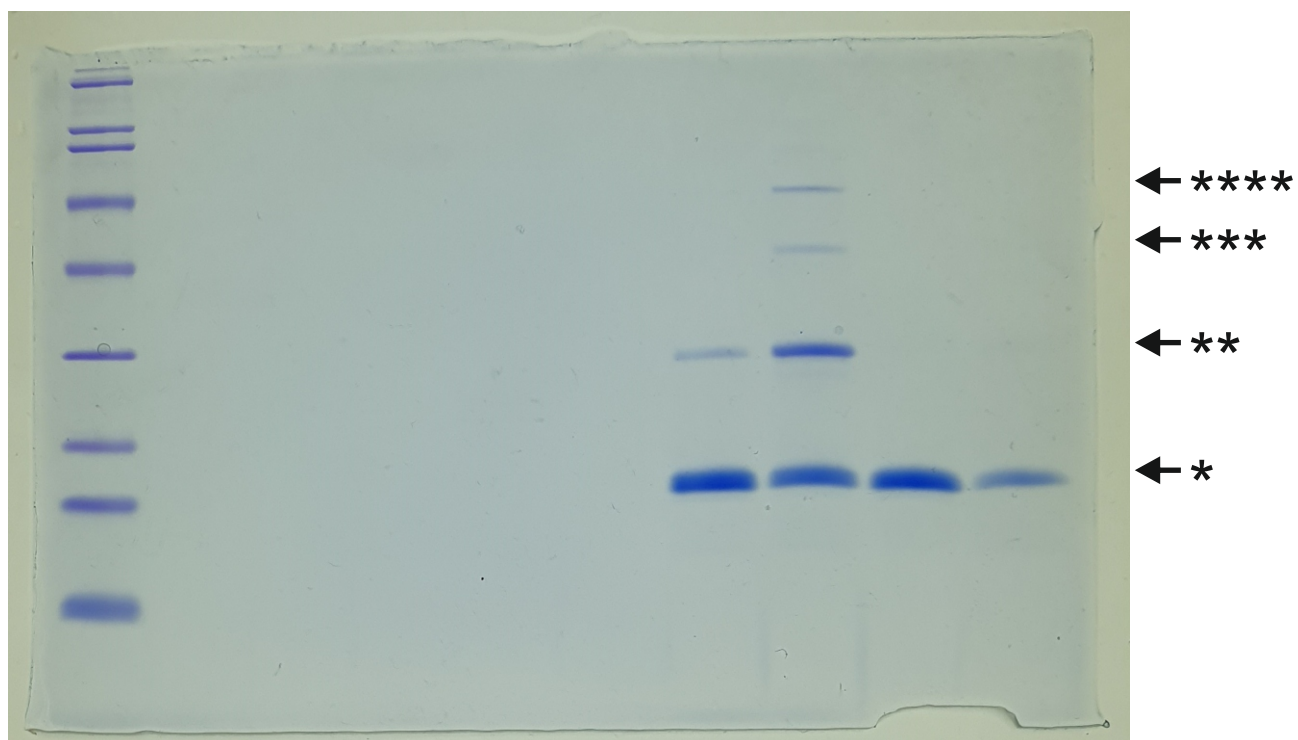

Supplement: Figure 2—source data 1. [file elife-93489-fig2-data1.zip › Figure 2- source data 1 Related to Figure 2B.pdf]

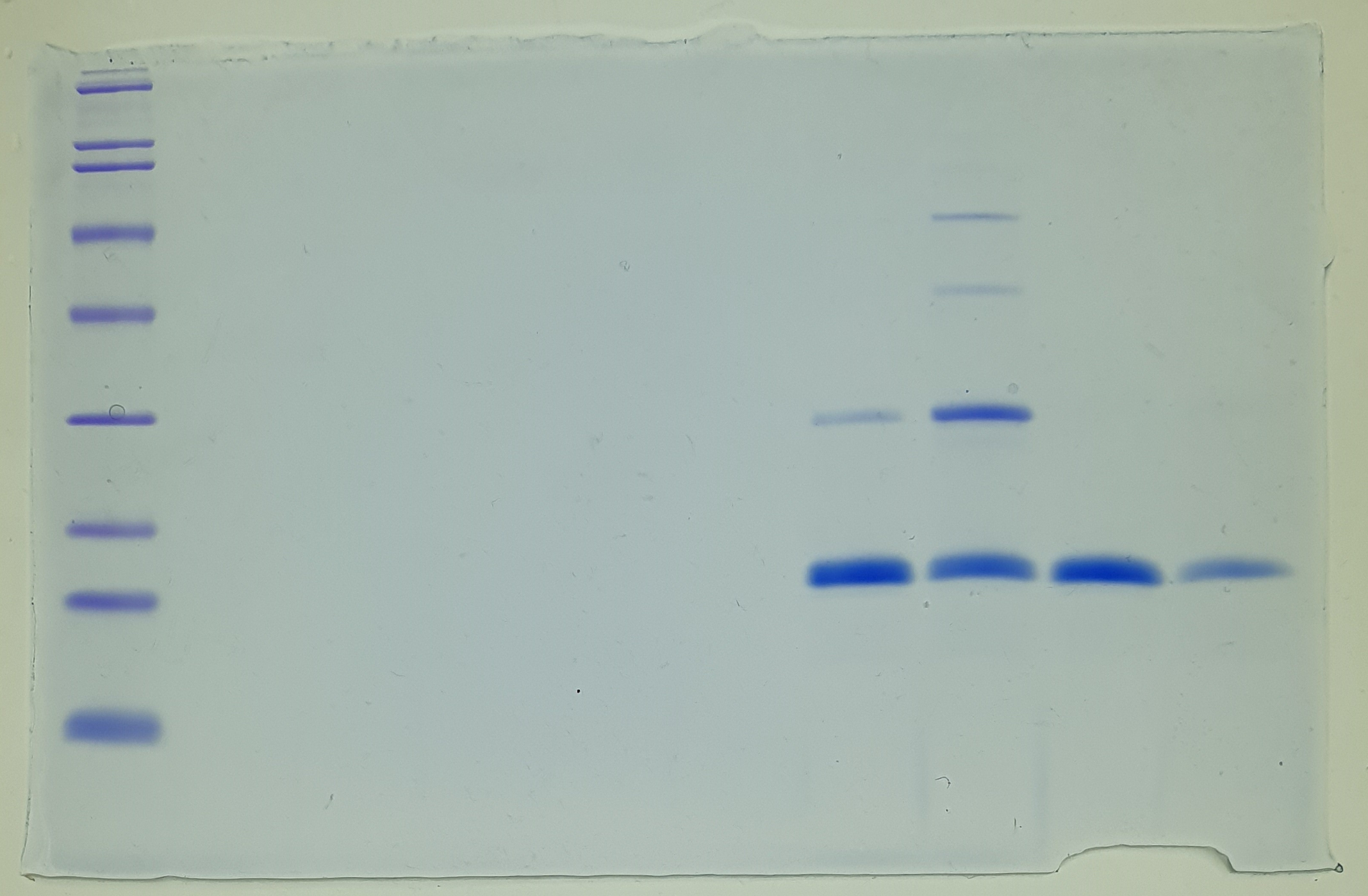

Supplement: Figure 2—source data 1. [file elife-93489-fig2-data1.zip › Figure 2- source data 2 Related to Figure 2B.tiff]

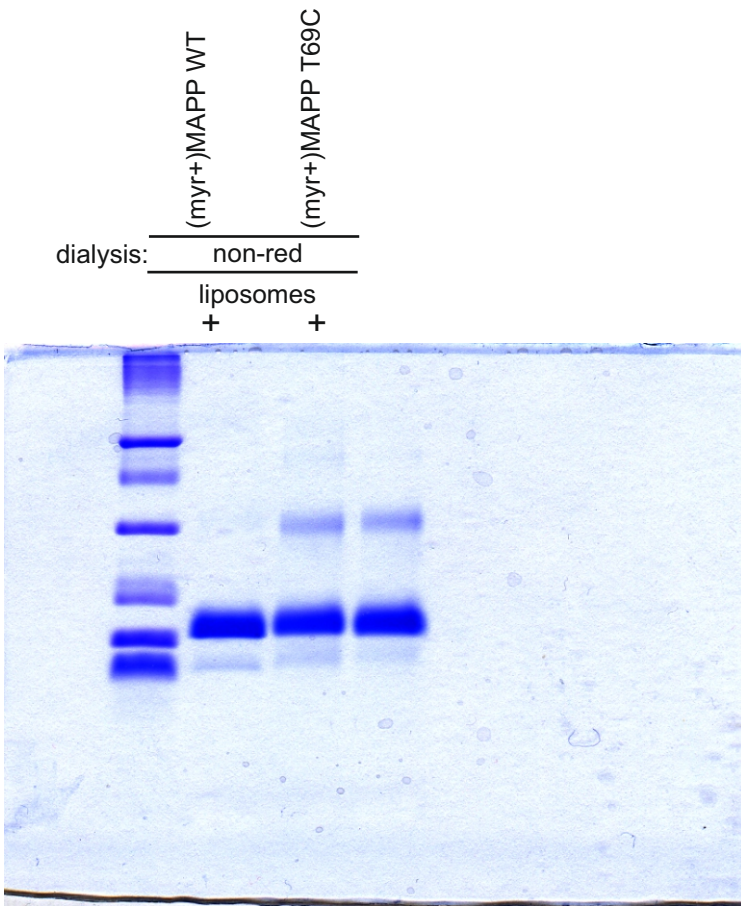

Supplement: Figure 2—figure supplement 1—source data 1. [file elife-93489-fig2-figsupp1-data1.zip › Figure 2- figure supplement 1 - source data 1.pdf]

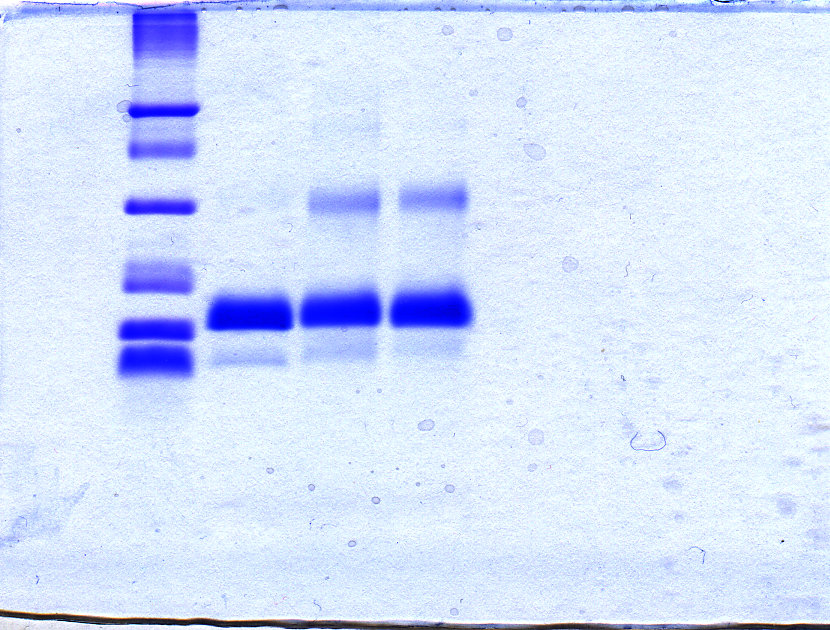

Supplement: Figure 2—figure supplement 1—source data 1. [file elife-93489-fig2-figsupp1-data1.zip › Figure 2- figure supplement 1 - source data 2.tif]
